# Functional Magnetic Resonance Imaging Neurofeedback-guided Motor Imagery Training and Motor Training for Parkinson’s Disease: Randomized Trial

**DOI:** 10.3389/fnbeh.2016.00111

**Published:** 2016-06-08

**Authors:** Leena Subramanian, Monica Busse Morris, Meadhbh Brosnan, Duncan L. Turner, Huw R. Morris, David E. J. Linden

**Affiliations:** ^1^MRC Centre for Neuropsychiatric Genetics and Genomics, School of Medicine, Cardiff UniversityCardiff, UK; ^2^Cardiff University Brain Research Imaging Centre, School of Psychology, Cardiff UniversityCardiff, UK; ^3^Trinity College Institute of Neuroscience, Trinity CollegeDublin, Ireland; ^4^Faculty of Psychology and Neuroscience, Maastricht UniversityMaastricht, Netherlands; ^5^Neurorehabilitation Unit, School of Health, Sport and Bioscience, University of East LondonLondon, UK; ^6^Department of Clinical Neuroscience, Institute of Neurology, University College LondonLondon, UK

**Keywords:** Parkinson’s disease, real-time functional magnetic resonance imaging, neurofeedback, motor training, WiiFit

## Abstract

**Objective**: Real-time functional magnetic resonance imaging (rt-fMRI) neurofeedback (NF) uses feedback of the patient’s own brain activity to self-regulate brain networks which in turn could lead to a change in behavior and clinical symptoms. The objective was to determine the effect of NF and motor training (MOT) alone on motor and non-motor functions in Parkinson’s Disease (PD) in a 10-week small Phase I randomized controlled trial.

**Methods**: Thirty patients with Parkinson’s disease (PD; Hoehn and Yahr I-III) and no significant comorbidity took part in the trial with random allocation to two groups. Group 1 (NF: 15 patients) received rt-fMRI-NF with MOT. Group 2 (MOT: 15 patients) received MOT alone. The primary outcome measure was the Movement Disorder Society—Unified PD Rating Scale-Motor scale (MDS-UPDRS-MS), administered pre- and post-intervention “off-medication”. The secondary outcome measures were the “on-medication” MDS-UPDRS, the PD Questionnaire-39, and quantitative motor assessments after 4 and 10 weeks.

**Results**: Patients in the NF group were able to upregulate activity in the supplementary motor area (SMA) by using motor imagery. They improved by an average of 4.5 points on the MDS-UPDRS-MS in the “off-medication” state (95% confidence interval: −2.5 to −6.6), whereas the MOT group improved only by 1.9 points (95% confidence interval +3.2 to −6.8). The improvement in the intervention group meets the minimal clinically important difference which is also on par with other non-invasive therapies such as repetitive Transcranial Magnetic Stimulation (rTMS). However, the improvement did not differ significantly between the groups. No adverse events were reported in either group.

**Interpretation**: This Phase I study suggests that NF combined with MOT is safe and improves motor symptoms immediately after treatment, but larger trials are needed to explore its superiority over active control conditions.

## Introduction

Parkinson’s disease (PD) is the second most common neurodegenerative disorder (de Lau and Breteler, 2006) affecting the motor circuit and affecting both motor and non-motor functions. PD currently affects about 5 million people aged over 50 worldwide and this figure is expected to rise to approximately 9 million over the next 25 years (Dorsey et al., 2007) because of increasing life expectancy. Diagnosis of PD is mainly clinical, although neuroimaging techniques are used for differential diagnosis (Politis, 2014). Pharmacotherapy with dopamine precursors (levodopa), dopaminergic (dopamine receptor agonists, COMT inhibitors, MAO-B inhibitors) or anticholinergic drugs is the mainstay of current treatments (Connolly and Lang, 2014) and gives most patients a considerable amount of motor symptom control. However, not all motor symptoms respond equally well, and cognitive decline and motor progression cannot be prevented. Furthermore, long-term treatment often leads to drug-induced dyskinesias and behavioral side effects (Hametner et al., 2010; Beaulieu-Boire and Lang, 2015). Deep-brain stimulation (DBS) is available as an invasive option to counteract tremor, rigidity and bradykinesia, but can result in psychiatric, neurological and surgical complications (Piasecki and Jefferson, 2004; Fenoy and Simpson, 2014). Various types of motor training (MOT) therapies and non-invasive brain stimulation are under development.

Real-time functional magnetic resonance imaging (rt-fMRI) neurofeedback (NF) has been used to self-regulate brain regions and modify behavior in healthy individuals and some early clinical work (review by deCharms, 2008; Caria et al., 2012; Weiskopf, 2012; Birbaumer et al., 2013). A number of motor related areas have been successfully modulated using motor imagery with fMRI NF, primary motor cortex (Yoo and Jolesz, 2002; deCharms et al., 2004; Yoo et al., 2008; Lee et al., 2009; Chiew et al., 2012), premotor cortex (Johnson et al., 2012) and supplementary motor cortex (Posse et al., 2001). Successful modulation of motor areas has also been shown to modulate functional motor networks (Hui et al., 2014; Xie et al., 2015).

In our previous proof of concept study (Subramanian et al., 2011) we compared five patients with PD who performed motor imagery with fMRI-NF and five patients who performed motor imagery in the scanner without receiving feedback. Only the first group showed an increase in activity in the target area the supplementary motor area (SMA) and in turn improved their motor functions. This study provided preliminary evidence that just being in the scanner environment and performing imagery was not sufficient for a change in motor functions but that NF was required. We have now developed an intervention that combines the concepts of brain stimulation and MOT and mental imagery practice.

It has been shown that motor imagery leads to activity in the SMA (Park et al., 2015), which is involved in the initiation and control of movements, has bidirectional connections with the basal ganglia (Lindenbach and Bishop, 2013) and has been reported to be underactive in PD (Nachev et al., 2008). Motor imagery can also induce motor cortical plasticity (Avanzino et al., 2015). Therefore using motor imagery to activate the SMA could lead to changes in the cortico-basal-ganglia circuit (affected in PD) inducing plasticity.

Motor imagery has been shown to improve motor performance, but the effect is short-lived and this may be due to lack of feedback (Driskell et al., 1994). Therefore by targeting the SMA and using motor imagery to induce activity in this area and providing immediate feedback on the performance, we may be able to produce more lasting changes in motor circuits, leading to improved motor performance. We therefore trained patients to upregulate the SMA through NF with fMRI signals (Subramanian et al., 2011).

Patients had an opportunity to transfer the mental imagery skills acquired during NF into the real life setting by practicing the motor imagery at home. Because motor imagery in combination with physical training has been shown to improve some motor symptoms in PD patients (Tamir et al., 2007) we added another transfer technology, using feedback-based motor exercise on a gaming console (the Nintendo Wii Fit device). The device when used for exercise training has been shown to reduce the severity of motor symptoms in PD patients (Barry et al., 2014). This intervention, which involved three NF training sessions, regular imagery homework and six exercise sessions was compared with a matched control intervention that only involved exercise on the gaming console in a randomized trial with blinded assessments.

Our challenge was to design an active control condition that was not already available as an established treatment protocol. Our rationale was as follows: the first 4 weeks of intervention were designed to compare the effect of NF and exercise separately to see if NF alone was better than exercise alone. As exercise has previously been shown to have some benefits in alleviating motor symptoms of PD, in the last 6 weeks of the intervention we added exercise to the NF training. We expected the NF training to have an additional benefit but included the exercise component as a cheaper and more widely available transfer technology.

## Materials and Methods

### Patients, Eligibility and Randomization

Thirty PD patients (27 males; 3 females) were recruited through PD specialist physicians and nurses from two NHS health boards and screened for eligibility at outpatient clinics. Two additional patients who were recruited and randomized did not start the intervention because they could not fit it into their work schedules. Participants received their usual medication (levodopa, dopamine agonists, amantadine, anticholinergics or monoamine oxidase B inhibitors), which did not change over the course of the study. The levodopa equivalent dose (Table 1) was calculated (Tomlinson et al., 2010) and used in the randomization procedure (Odekerken et al., 2013; two levels: above and below 600 mg) along with the age of the patient (two levels: 65 and above and below 65), to assist in group matching. Randomization was overseen by the South East Wales Clinical Trials Unit using the method of minimization.

**Table 1:** **Baseline characteristics**.

Demographics (all patients)	NF group (*N* = 15) Mean ± SD	MOT group (*N* = 15) Mean ± SD	*p*-value
Gender (male/female)	14/1	12/3	−
Hoen and Yahr stage (I/II/III)	7/7/1	5/10/0	−
Age (years)	67 ± 9	63 ± 11	0.6
Time since diagnosis (months)	51 ± 38	57 ± 33	0.6
LEDD	456 ± 219	599 ± 418	0.6
MoCA	26.3 ± 2.5	26.7 ± 1.8	0.6
**Included in final analysis**:	***N* = 13**	***N* = 13**	
Primary outcome measure (Off Med)
MDS-UPDRS-MS	23.3 ± 9.4	26.7 ± 12.6	0.9
Secondary outcome measures (On Med)
MDS-UPDRS-MS	22.9 ± 7.5	21.7 ± 8.0	0.9
MDS-UPDRS-M-DL	13.1 ± 6.9	13.2 ± 7.3	0.9
MDS-UPDRS-NM-DL	9.8 ± 3.9	9.5 ± 4.6	0.9
MDS-UPDRS-SS-DL	49.5 ± 15.6	48.7 ± 18.9	0.9
PDQ-39	19.4 ± 10.4	24.4 ± 15.6	0.9
**Quantitative assessments (On Med)**
Actigraph (Average steps)	3856 ± 2352	4277 ± 2317	0.9
GaitRite-Walking speed (m/s)	1.1 ± 0.2	1.1 ± 0.2	0.9
GaitRite-Walking cadence	114 ± 9	111 ± 10	0.9
(total steps/min)			
GaitRite-Walking step	59 ± 11	60 ± 10	0.9
length left (cm)
GaitRite-Walking step	61 ± 10	60 ± 11	0.9
length right (cm)

*NF, neurofeedback; MOT, motor training; SD, standard deviation; N, total number of patients; LEDD, Levodopa equivalent dose; MoCA, Montreal Cognitive Assessment scale; Med, medication. P-values are FDR corrected*.

Patients were eligible to take part if they had a diagnosis of PD (according to the UK PD Brain Bank Clinical Diagnostic Criteria), disease severity within Hoehn and Yahr stages I-III, no dementia (cut-off score <21/30 on the Montreal Cognitive Assessment Scale (MoCA; Dalrymple-Alford et al., 2010) or significant comorbidity and fulfilled the safety requirements for MRI.

### Standard Protocol Approvals, Registrations and Patient Consents

The study was approved by the South East Wales Research Ethics Committee, the Cardiff School of Psychology Ethics Committee, Aneurin Bevan and Cardiff and Vale Health Boards. All participants gave written informed consent to participate in the study in accordance with the Declaration of Helsinki. The trial was registered with Clinicaltrials.gov (NCT01867827) in May 2013 before the first patient completed intervention. Enrollment was between the end of March 2013 and October 2013 and the last patient completed intervention at the end of January 2014.

### Design

**Figure 1:**
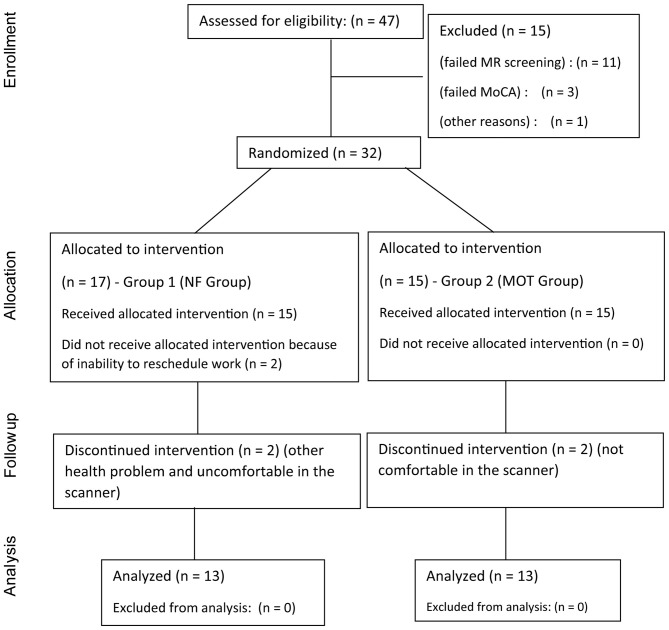
**The CONSORT diagram shows the flow of patients through each stage of the randomized, controlled trial**.

**Figure 2:**
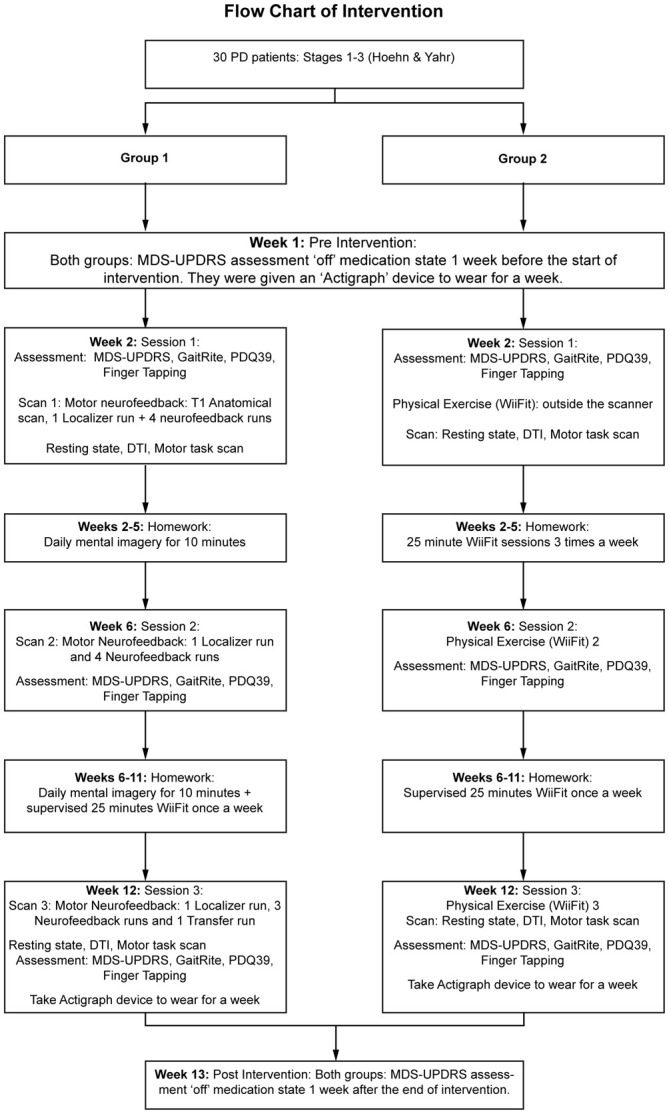
**Flowchart of the study design with details of the interventions**.

This was a small safety and efficacy randomized controlled trial testing two different interventions (Figure 1: CONSORT diagram). Group 1 (NF) received rt-fMRI-NF (three sessions: at the start of the intervention in week 2 (PRE; session 1), week 6 (session 2) and at the end of the intervention in week 12 (POST; session 3) and regular homework employing mental imagery for the first 4 weeks of intervention followed by 6 weeks of supervised MOT with a virtual reality gaming device (Nintendo Wii) during the rest of the intervention period. Group 2 (MOT) received supervised MOT on the gaming device throughout the full intervention period of 10 weeks. No changes were made to the protocol once the trial had commenced. The study procedure is summarized in Figure 2.

The sample size of 15 in each group who started the intervention is similar to randomized controlled trials in PD with intense exercise therapy (Uhrbrand et al., 2015) and other rt-fMRI-NF studies (Linden et al., 2012). We wished to document safety and efficacy of the interventions and to calculate indices of patient adherence and attrition. Furthermore, we wished to estimate the sample size for a full powered random, controlled trial testing the potential benefit of rt-fMRI-NF over and above MOT without NF.

### Primary Clinical Outcome Measure

The motor examination of the MDS-UPDRS (Goetz et al., 2008) was used as the primary clinical outcome measure and administered PRE and POST intervention when patients were in their “off medication” state and also videotaped for later evaluation. Patients were requested not to take their medication for 12 h before assessment. The examination was carried out by two raters (authors LS and DEL), and the inter-rater reliability for each item was high with an inter-class correlation of 0.94 (pre-assessment) and 0.92 (post-assessment). Scores provided by the blind rater (DEL) were used in the analysis. The off-medication assessments were done 1 week before (Pre) and 1 week after (Post) the intervention, generally as part of home visits that also entailed setting up/collecting the actigraphy equipment.

### Secondary Outcome Measures

Patients were tested on these measures at three time points (PRE intervention, after 4 weeks intervention and POST intervention at 10 weeks) in the “on medication” state.

The MDS-UPDRS full scale measured non-motor experiences of daily living (NM-DL), motor experiences of daily living (M-DL) and motor complications in addition to the motor examination (motor scale, MS) (which was videotaped for later evaluation). The Parkinson’s Disease Questionnaire-39 (PDQ-39) measured different aspects of PD and quantitative motor measures were provided by the Actigraph GT3X and GaitRite (see Table 1).

The PDQ-39 is self-administered and measures activities of DL, emotional well-being, stigma, social support, cognitive impairment, communication and bodily discomfort.

The Actigraph GT3X activity monitor (ActiGraph, LLC, Pensacola, FL, USA) is a device worn on the waist to measure daily activity levels. The software generates a clinical report of measures such as number of steps taken during the time the device was worn, the lifestyle, how sedentary or vigorous the participant was and how much energy was expended. Wetten et al. (2014), show that the Actigraph is a valid tool for quantifying energy expenditure during light intensity stepping. The number of steps taken was compared between the PRE- and POST-intervention assessments, when the device was worn for a week during each assessment.

Changes in gait patterns were measured with the GaitRite (CIR Systems/GAITRite^®^, CIR Systems Inc., Franklin, NJ, USA). This electronic gait analysis system uses a carpet with sensor pads on it to measure quantitative kinematic parameters of gait such as speed, number of steps and step length. Patients were required to walk five times up and down the carpet to obtain their gait measures at each of the three assessment sessions in weeks 2, 6 and 12. Data was automatically generated by the GaitRite software and comparison between the two groups was performed using the SPSS statistical package.

We also looked at attrition rate (Figure 1), adherence to the protocol and adverse events. An acceptable attrition rate, based on similar complex interventions, would be 25% (Stack et al., 2012). Because of the relatively unique nature of our homework design there were no benchmarking figures, but we would assume protocol adherence of at least 75% to be required for successful transfer of learnt skills into everyday settings based on existing literature (Hlavaty et al., 2011). Adverse events were defined as any subjective complaints of physical or mental deterioration or any physical or psychological impairment requiring medical intervention.

### Screening before Exercise Sessions

All patients filled in the Physical Activity Readiness Questionnaire PAR-Q before starting the exercise sessions in order to determine the safety to carry out physical exercise. In addition their blood pressure and heart rate were measured before each exercise session which went ahead only if the values were within the normal range.

### NF Group: Neurofeedback Protocol

Each NF scanning session consisted of a localizer run and four NF runs, with the exception that in session 3 the fourth run was a transfer run to see if patients were able to achieve reliable upregulation without feedback. The localizer run allowed us to identify the target area (SMA). Activity in this area was then used to give feedback to the patients during the NF runs.

During the localizer run participants were presented with an image of a thermometer (with 10 levels) in the center of a colored background. They were required to execute a motor task involving sequentially pressing their thumb to each of the other fingers for 20 s when the background was green and rest for 20 s when the background turned yellow. The run had nine cycles of four motor execution and five rest and lasted 3 min.

This simple motor task has been shown to reliably activate motor networks (Gordon et al., 1998), including the SMA that we aimed to target (Gerloff et al., 1997). We computed movement related activation maps online using the TurboBrainVoyager software package in real-time.

The localizer run was followed by four NF runs (3 min each). The procedure is similar to the motor execution localizer run in terms of timing but here, instead of moving their digits, the patients had to increase activity in the target area through motor imagery only. They received continuous feedback about the level of activation through the thermometer display, which was controlled by their own brain activity in the SMA target area through a brain computer interface that coupled the output of the rt-fMRI analysis with the control of the stimulus display. As activity in the SMA target area increases, the bars of the thermometer filled with red. Patients were informed that they might be able to increase activity in the SMA through motor imagery, but were free to use any specific imagery strategy. We did not prescribe a particular imagery strategy, because we considered the adaptive development of the optimal imagery strategy to be one of the key components of NF.

In the first 4 weeks of the intervention, patients in the NF group were asked to practise motor imagery (strategies they had used during NF) at home for 10 min every day and keep a record. After the first 4 weeks, they carried on with the imagery homework and in addition came in once a week for 25 min of MOT on the Nintendo Wii.

### fMRI: Data Acquisition

Functional and anatomical scans were performed in a 3 Tesla GE (General Electric) MRI scanner at the Cardiff University Brain Research Imaging Centre. A high-resolution T1-weighted anatomical scan (178 slices) covering the whole brain was acquired using a fast spoiled gradient-recalled-echo (FSPGR) pulse sequence. Functional data was obtained using a single-shot EPI sequence (TR, 2 s; TE, 35 ms; 30 slices; 3 mm thick; flip angle, 80°). The first six volumes were discarded to allow for T1 equilibration effects. A total of 100 volumes were acquired in each functional run for each participant. Scans were acquired in the ascending order and interleaved.

### Cameras

Four Qualisys Cameras situated in the MR suite were used to monitor any tremors in the hands. Two markers were positioned on the tremor dominant hand/dominant hand of the patients to measure any change in movement during the NF runs. The movement of the markers was captured 120 times every second and the raw data (movement of the hand in millimetres) was extracted for each run separately. The data frequency was reduced to blocks of 20 s allowing us to differentiate between the resting and imagery periods of the run. The data points were then subtracted from the baseline (starting position of the hand) to obtain an indication of how much the hand had moved. Because of time constraints this information was only available for eight sessions. The average movement during the resting and imagery conditions in the NF runs was negligible (Resting = 0.28 mm and Imagery = 0.35 mm).

### MOT Group: Motor Training

Patients in this group carried out a simple MOT programme on the Nintendo Wii for three separate sessions in week 2, 6 and 12. The task was to step on and off the balance board and sideways following the pattern on the computer screen, and continuous performance feedback was provided by an avatar.

In the first 4 weeks patients also came into the laboratory three times a week for 25 min of MOT on the gaming device. After the first 4 weeks, they continued with the MOT, but now only once a week for 6 weeks.

### Both Groups

#### Details of Motor Training

During the weekly training sessions in weeks 6–12, the motor task was to step on and off the virtual reality gaming device balance board keeping in time to a metronome or following an avatar on the screen. Each session consisted of two 10 min blocks and one 5 min block, interleaved with rest periods. Patients could also pause and rest at any time during the training.

Both groups were matched for intervention time in the first month (~5 h) and for contact time in the following 6 weeks (3 h). In addition to this the NF group had 10 min of daily motor imagery practise at home.

Participants in both groups underwent a half hour scan session before and after the intervention with resting state fMRI, diffusion tensor imaging and fMRI during a simple motor task (results from these scans are not reported here). Therefore all patients who took part in the study were suitable and experienced being in a scanner environment.

### Statistical Analysis

The primary and secondary outcome measures were analyzed using the SPSS statistical package (version 20).

An independent samples *t*-test was performed on all the measures to determine any difference between the groups at baseline (Table 1).

A paired samples *t*-test was carried out to determine changes in the outcome measures from PRE to POST intervention within each group. We also performed analysis of covariance (ANCOVA) on all the measures at POST intervention with the PRE intervention score as the covariate to test for any significant difference between the groups (Table 2).

**Table 2:** **Changes from baseline to after intervention within and between groups**.

	NF group Δ Mean ± SD	*P* (ES)	MOT group Δ Mean ± SD	*P* (ES)	ANCOVA (ΔNF vs. ΔMOT) *p*
**Primary outcome measure** (Off Med)
MDS-UPDRS-MS	−4.5 ± 3.3	0.00 (0.8)	−1.8 ± 8.3	0.48	0.73
**Secondary outcome measures** (On Med)
MDS-UPDRS-MS	−4.9 ± 3.8	0.000 (0.8)	−5.4 ± 4.9	0.02 (0.8)	0.86
MDS-UPDRS-M-DL	−1.7 ± 2.3	0.04 (0.6)	−1.5 ± 2.8	0.24	0.86
MDS-UPDRS-NM-DL	−2.8 ± 2.9	0.01 (0.7)	−0.9 ± 3.9	0.48	0.73
MDS-UPDRS-SS	−9.2 ± 9.7	0.01 (0.7)	−7.9 ± 8.4	0.02 (0.7)	0.86
PDQ-39	−2.4 ± 4.8	0.16	−3.6 ± 6.5	0.24	0.93
**Quantitative assessments**
Actigraph (Average steps)	487 ± 1270	0.29	252 ± 928	0.48	0.86
GaitRite-Walking speed	0 ± 0	0.88	0 ± 0	0.39	0.81
GaitRite-Walking cadence	0 ± 7	0.88	1 ± 7	0.66	0.86
GaitRite-Walking step length left	0 ± 4	0.88	2 ± 6	0.39	0.81
GaitRite-Walking step length right	−1 ± 4	0.78	2 ± 6	0.39	0.73

*NF, neurofeedback; MOT, motor training; SD, standard deviation; ES, effect size; Med, medication. P-values are FDR corrected*.

A repeated measures analysis of variance (ANOVA) with time (sessions 1, 2, 3) as within subject and group (NF vs. MOT) as between subject factors on the secondary outcome measures was also performed to test for time-dependent changes in variables and interaction between time and group factors (Table 3). Multiple comparisons of tests was accounted for by adjusting the false discovery rate (FDR) using the “R” statistical package (version 3.2.4).

**Table 3:** **ANOVAs for clinical secondary outcome measures “on-medication” across all three time assessments**.

	ANOVA		
Measures	Effect of time	Effect of group	Time × Group interaction effect
MDS-UPDRS-MS	*F*_(2,48)_ = 8.36, *p* = 0.002 (ES = 0.26)	*F*_(1,24)_ = 0.15, *p* = 0.7 (N/S)	*F*_(2,48)_ = 3.51, *p* = 0.03 (ES = 0.13)
MDS-UPDRS-SS	*F*_(2,48)_ = 13.46, *p* = 0.000 (ES = 0.36)	*F*_(1,24)_ = 0.32, *p* = 0.7 (N/S)	*F*_(2,48)_ = 7.64, *p* = 0.005 (ES = 0.24)
MDS-UPDRS-M-DL	*F*_(2,48)_ = 4.34, *p* = 0.01 (ES = 0.15)	*F*_(1,24)_ = 0.22, *p* = 0.7 (N/S)	*F*_(2,48)_ = 5.58, *p* = 0.01 (ES = 0.19)
MDS-UPDRS-NM-DL	*F*_(2,48)_ = 4.98, *p* = 0.01 (ES = 0.17)	*F*_(1,24)_ = 0.55, *p* = 0.7 (N/S)	*F*_(2,48)_ = 2.14, *p* = 0.13, (N/S)
PDQ-39	*F*_(2,48)_ = 4.46, *p* = 0.02 (ES = 0.16)	*F*_(1,24)_ = 1.91, *p* = 0.7 (N/S)	*F*_(2,48)_ = 3.79, *p* = 0.03 (ES = 0.14)

*ANOVA, Analysis of variance; MDS-UPDRS, Movement Disorder Society-Unified Parkinson’s Disease Rating Scale; MS, motor subscale; SS, summed score; M-DL, Motor aspects of daily living; NM-DL, non motor aspects of daily living; PDQ, Parkinson’s disease questionnaire; ES, effect size; N/S, not significant; *P*-values are FDR corrected*.

#### fMRI Data Analysis (On-Line)

Data from the motor execution localizer and motor imagery NF runs were obtained from the scanner and analyzed online on an analysis computer (Johnston et al., 2010) before being fed back to the participant in the scanner. The real-time fMRI software package, TurboBrainVoyager version 3.2 (TBV, Brain Innovation) was used. Data was read volume by volume, corrected for angular and translational motion and analyzed with an incremental general linear model (GLM). For the motor execution localizer run, we used one predictor for the movement blocks (convolved with a canonical hemodynamic response function) to identify areas of movement-related activation. The *t-*value for the contrast between movement and rest in the localizer map was set at 3. We took the peak midline activation from the SMA during the motor execution localizer run as the target area for the NF runs. The region of interest was selected by choosing the whole of the extent in the *x–y *plane (on the axial slice) encompassing the activity in the SMA with an extent across three slices in the *z* direction.

In the NF runs, the “thermometer” displayed the percentage signal change to patients (with a full thermometer denoting 1% signal change, which is around the maximum of what patients could achieve) from baseline computed for the top third of most active voxels of the region of interest for an average of three time points. The baseline value was set to the average signal intensity value recorded from the last three time points during the preceding “fixation” period to the current upregulation block. Patients were informed of the approximate 5 s time lag between a change in neural activity and its reflection in the blood-oxygen-level dependent signal (and thus on the feedback display). The stimulation interface was custom programmed in PsychoPy (Peirce, 2007) and presented from a Macintosh computer. This interface allows updating of feedback within ~1 s of data acquisition.

#### fMRI Data Analysis (Off-Line)

We conducted further off-line fMRI analyses to ascertain whether participants reliably upregulated the target area (region-of-interest [ROI] analysis) and whether activation patterns changed as a result of the NF intervention in other parts of the brain (whole brain analysis).

fMRI data were preprocessed using the BrainVoyager QX software package (version 2.6; Brain Innovation, Maastricht, Netherlands). Data for each subject was analyzed using a GLM with one predictor for the “motor execution” condition in the localizer and a single predictor for “upregulation” during the motor imagery NF runs. The regressors for both were convolved with a canonical hemodynamic reference function accounting for the temporal delay and dispersion of the hemodynamic response. Six motion confounds and two for heart rate and respiration were added to the GLM for each of the runs. The models were used for both ROI and whole brain random effects group analysis.

Motion correction and temporal filtering were performed on the raw data to remove artifacts due to head movement and physiological noise. The functional data were then co-registered to the T1 anatomical scan and transformed into Talairach coordinate space (Talairach and Tournoux, 1998). Spatial smoothing with a Gaussian filter (full width at half maximum (FWHM) of 6 mm) was applied to the volume time course files.

For the ROI analysis we extracted the beta and *t*-values for the “upregulation” predictor from the SMA target area for each run and subjected them to a repeated measures ANOVA with the within-subject factor of session. We performed a correlation analysis (Pearson’s r) between the improvement of the MDS-UPDRS clinical scores (Pre-Post) and the overall NF success (measured in average *t*-value across the three sessions).

For the whole brain analysis an average of the participant’s anatomical scans was created and used in the analysis. A whole brain mask was also created to restrict the number of voxels used. We used the cluster-level correction approach implemented in the Brainvoyager software (1000 iterations) to control for multiple comparisons across the brain (cluster-level corrected threshold: *p* < 0.05).

#### Whole Brain Analysis

We conducted a random effects analysis in Brain Voyager, for activation during the upregulation sessions in the 10 participants with full data sets (2 sessions with 4 runs each and 1 session with 3 runs; transfer run was excluded) corrected for multiple comparisons across the brain with cluster level correction.

## Results

### Sample Characteristics

Data were approximately normally distributed. Shapiro-Wilk’s test was non-significant (*p* > 0.05) for all measures except the scores of the MDS-UPDRS MS “on medication” (MOT group) sessions 2 and 3 and MDS-UPDRS NM-DL (NF group) session 1. For these measures visual inspection of Q-Q plots confirmed approximately normal distribution.

### Primary Outcome Measure: PRE- vs. POST-Intervention

**Figure 3:**
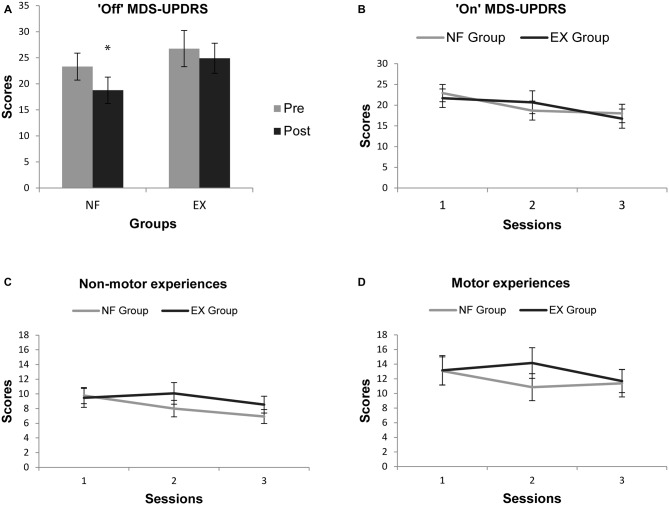
**(A)** “Off- medication” PRE- and POST-intervention mean scores of the Movement Disorder Society-Unified Parkinson’s Disease Rating Scale (MDS-UPDRS) motor subscale (MS) for both groups with higher scores indicating greater impairment; **(B)** “On-medication” mean scores of the MDS-UPDRS MS for the three assessment sessions for both groups with higher scores indicating greater impairment; **(C)** “On-medication” mean scores of the non-motor experience of daily living (NM-DL) sub scale of the MDS-UPDRS for the three assessment sessions for both groups; **(D)** “On-medication” mean scores of the motor-experience of daily living sub scale of the MDS-UPDRS for the three assessment sessions for both groups. * indicates significant difference.

The ANCOVA analysis revealed no statistically significant difference between the effects of the intervention in the NF vs. MOT groups (*F*_(1,23)_ = 2.62; *p* = 0.11; Table 2). Participants in the NF group improved on average by −4.5 points on the MDS-UPDRS-MS-off-medication (95% confidence interval −2.5 to −6.6), whereas those in the MOT group only tended to improve by −1.9 points (confidence interval +3.2 to −6.8; see Table 2 and Figure 3A).

### Secondary Outcome Measures: PRE- vs. POST-Intervention

The ANCOVA’s showed no significant difference between the groups in any of the measures (Table 2). Results of the paired samples *t*-test and ANCOVAs on all the clinical and quantitative measures are reported in Table 2.

### Time-Dependent Changes in Secondary Clinical Measures During the Interventions

Results of the ANOVAs on the “on-medication” clinical measures acquired at the three assessment time points are reported in Table 3. Of these, the MDS-UPDRS-MS, MDS-UPDRS-SS, MDS-UPDRS-M-DL and PDQ-39 showed a significant interaction term for time × group analysis, driven by faster improvements in the NF group. Two of the time × group interactions (on MDS-UPDRS-SS and MDS-UPDRS-M-DL) survived Bonferroni correction for multiple testing (across the five clinical secondary outcome measures) at a corrected *p*-value <0.05.

### Adherence

Twenty six of the 30 patients who underwent the interventions completed the study, which yields an attrition rate of 13% (Figure 2). Patients in the NF group adhered to the imagery homework (11 of 13 patients returned the diaries at the end of the study), with a 75% rate of completed homework (calculated as daily entries). For the MOT group, the adherence rate was 84%. No adverse events were reported in either group.

### Functional Magnetic Resonance Imaging Analysis: ROI Analysis

Patients in the NF group were able to upregulate the SMA during the NF sessions. Successful upregulation was defined as a positive “*t*” or “beta” value for the upregulation vs. baseline contrast from the brain voyager analysis.

**Figure 4:**
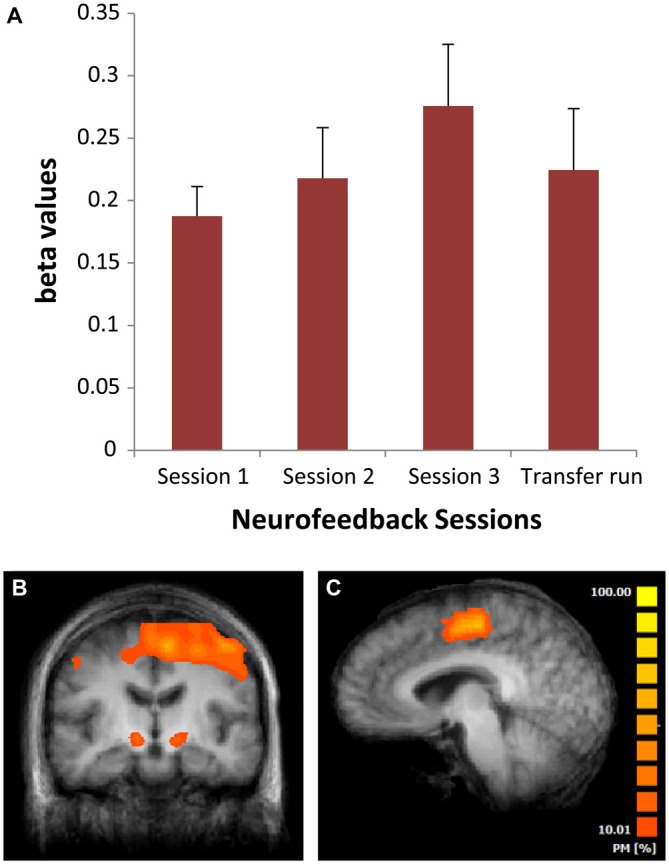
**(A)** Mean beta values from the neurofeedback (NF) scans for the three different sessions in weeks 2, 6 and 12 of the NF intervention. Bars are standard errors; **(B)** Areas of significant activation across all NF sessions (whole brain based analysis). **(C)** Sagittal view of the brain showing a probabilistic map of the overlap across regions of interest (ROI’s) supplementary motor area (SMA) used for NF training across patients. PM, probabilistic map.

Using SPSS we computed a one way repeated measures ANOVA with session as a factor. There was no significant increase in SMA activation across sessions (betas: *F*_(2,24)_ = 1.34; *p* = 0.28; Figure 4A; *t*-values: *F*_(2,24)_ = 1.19; *p* = 0.31), but there was a significant difference of the means from zero as seen from the intercept (betas: *F*_(1,12)_ = 21.60; *p* = 0.001; *t-values:* *F*_(1,12)_ = 20.47; *p* = 0.001), indicating successful SMA upregulation from baseline activity.

We found a trend-level correlation [*r* = −0.448, *p* = 0.06 (1-tailed)] between the improvement of the MDS-UPDRS clinical scores and the overall NF success.

Patients were able to upregulate the SMA without any change in voluntary or involuntary hand movements as measured using the camera system.

### Whole Brain Analysis

The whole brain analysis of activation during SMA upregulation blocks across all sessions revealed additional significant activity compared to baseline in the subthalamic nucleus, cerebellum, frontal areas, insula, putamen and anterior cingulate (Table 4 and Figure 4B).

**Table 4:** **Brain areas activated and deactivated during the NF runs from all sessions**.

Clusters of brain areas activated	Peak x/y/z	*t*-value	*p*-value	Cluster size (mm^3^)
Supplementary motor area	−7/1/57	7.98	0.000022	7434
Cingulate gyrus	5/7/27	3.62	0.005538	1678
Right insula	29/28/6	3.84	0.003911	4013
Left putamen	−28/−5/3	2.61	0.028177	200
Right globus pallidus	12/−7/−3	3.39	0.007986	257
Left globus pallidus	−16/−8/−3	3.16	0.011523	335
Right subthalamic nucleus	11/−11/−3	3.98	0.003186	650
Left subthalamic nucleus	−13/−11/−3	3.22	0.010461	423
Midbrain	−1/−17/−9	3.77	0.004388	1220
Cerebellum	35/−53/−24	7.68	0.000030	5166
Right parietal cortex	48/−62/30	−5.08	0.000659	4540
Left occipital cortex	−28/−86/−6	−5.30	0.000489	2453
Right occipital cortex	32/−83/0	−3.97	0.003215	628

## Discussion

We showed that a new intervention for PD that combines fMRI-based NF with mental imagery and MOT was safe and brought improvement of motor symptoms. The NF group showed an improvement of −4.5 points (−20%) on the primary clinical outcome measure of the “off-medication” MDS-UPDRS-MS. We used the off medication state motor assessment as the primary outcome measure because non-pharmacological interventions that promote compensation may operate through non-dopaminergic mechanisms (Bezard et al., 2003), and thus difference is most prominent in the off medication state. This clinical improvement was in the range of the minimal clinically important difference (−5 points; Schrag et al., 2006; Shulman et al., 2010) and similar to that seen in the NF group of our pilot study (Subramanian et al., 2011). It is also similar to that commonly achieved by adding a second drug to a dopamine agonist (decrease of up to 5 points on the UPDRS motor scale (MS); Pinter et al., 1999; Stocchi et al., 2004) and to that reported in a meta-analysis of transcranial magnetic stimulation (TMS) studies (pooled mean difference of −6 points on the UPDRS motor scale; Fregni et al., 2005).

Electroencephalography (EEG) based NF has been studied in PD patients with inconsistent findings and various limitations (Esmail and Linden, 2014). With fMRI based NF showing promising outcomes, transferring this learning to EEG NF could further maintain successful motor imagery (Bai et al., 2014) and induce brain changes (Ros et al., 2010) outside the scanner as this would be more cost effective in the long term. Recent studies have shown the possibility of identifying cortical signatures (Zich et al., 2015) and developing fMRI based EEG fingerprints for sub-cortical brain areas (Keynan et al., 2016) with simultaneous fMRI-EEG acquisition.

The intervention had a relatively high intensity in both groups and resulted in a clinically significant improvement in the NF group. However, the ANCOVA showed no significant group difference in the primary outcome measure or in the secondary clinical outcome measures of motor and non-motor functions or in the quantitative measures. Thus, we did not demonstrate statistical superiority for the NF compared to the MOT intervention, perhaps because MOT interventions can have therapeutic benefits in their own right (Barry et al., 2014; Shen and Mak, 2015). However, the present study can provide power estimates for future studies addressing the difference in efficacy between NF and active control interventions. Using the data from this pilot study to estimate the common standard deviation (SD) of 13 points for the MDS-UPDRS measure (the highest of the group SDs and therefore the most conservative) and setting the minimal clinically important difference as −5 points, for a power of 80% a two tailed Student’s *t*-test (α = 0.05) gives a required sample size of 101 per group in a two group comparison.

Although the secondary clinical outcome measures were similar across the two intervention groups at the POST-intervention assessment, the improvement may have occurred at an earlier stage of the intervention period (already at assessment time point 2) in the NF group (Figures 3B–D; statistics Table 3), as indicated by the significant group × time interaction for MDS-UPDRS-SS and MDS-UPDRS-M-DL. One explanation could be that motor skill acquisition was accelerated in the NF group, but further mechanistic studies would be needed to confirm this.

Only the NF group showed significant improvement on the “off-medication” UPDRS-MS, whereas both groups improved on the “on-medication” MDS-UPDRS-MS assessment. One possible reason might be that NF training leads to restitution of some aspects of basal ganglia (BG) function, which may confer benefits even in the absence of externally enhanced dopaminergic activity. However, because the majority of patients for whom NF is considered will likely be and remain on medication, motor assessment during the “on medication” state should be included as a primary outcome in future trials.

Although the correlation between the improvement of the MDS-UPDRS clinical scores and the overall NF success was not significant due to the small sample size, the association goes in the expected direction (the MDS-UPDRS clinical score and *t*-values are negatively correlated, thus improvement in the clinical scores was associated with higher activation of the target area). This preliminary indication of an association between the intervention and clinical improvement corroborates the need for larger, sufficiently powered studies of NF in PD.

The whole-brain imaging analysis suggested some mechanisms through which self-regulation training of the SMA may influence brain networks relevant in PD. During NF, we observed co-activation of other cortical motor areas and subcortical areas, including the subthalamic nucleus and putamen. This may suggest that self-regulation training of higher cortical motor areas can indeed modulate the subcortical loops implicated in PD, although more detailed analysis of NF imaging data, including functional connectivity analysis (Rota et al., 2011; Scharnowski et al., 2014) will be needed to probe any neuroplastic changes.

### Limitations

Our study did not have a control group in the scanner receiving sham feedback or any other control feedback. rtfMRI NF has been tested in other clinical applications such as chronic pain, tinnitus, stroke, depression, schizophrenia, obesity and addiction with some preliminary promising results with various control conditions and it is recognized that no single control condition can address all the mechanisms that may underlie clinical improvement under NF (Sulzer et al., 2013). We did not use sham feedback as this may frustrate patients and artificially induce superior effects in the real NF group. Instead we used an active control group with feedback based physical intervention, which was matched with the NF intervention for intensity.

With regard to the imagery strategy used by the patients, we did not prescribe a particular imagery strategy, because we considered the adaptive development of the optimal imagery strategy to be one of the key components of NF. Future studies could explore separately the effect of visual and kinesthetic motor imagery and differentiate between upper and lower limb imagery.

Because we did not demonstrate statistical superiority for the NF over MOT intervention in our sample one might query whether relatively expensive, laboratory-based interventions should be pursued, as compared to, for example, physiotherapy. However, in a recently completed randomized controlled trial of PD rehabilitation (Clarke et al., 2014) a combination of occupational therapy and physiotherapy was not found to be better than no therapy. Similarly, the MOT group in our study showed no relevant clinical improvement. Thus, there is a case for larger studies of NF-based interventions that will explore whether this technique can provide the crucial boost to other training-based interventions that leads to lasting clinical and functional improvements.

## Conclusion

rt-fMRI-NF is a new safe technique for self-regulation of motor networks in PD with good feasibility and patient adherence and preliminary evidence for short-term efficacy. However, further studies are needed to demonstrate its efficacy as an add-on treatment and superiority over other interventions. Inclusion of follow-up assessments would track the long-term retention of clinical improvements and enable the assessment of the cost-effectiveness of the NF intervention. Future trials may also help to identify neuro-imaging biomarkers for prediction of successful application of NF in individual PD patients.

## Author Contributions

LS, DEJL: study concept and design, drafting/revising the manuscript for content, analysis and interpretation of data. MBM, DLT, HRM: study concept and design, drafting/revising the manuscript for content. MB: statistical analysis of data, drafting/revising the manuscript for content. All authors have approved the final version of the manuscript.

## Conflict of Interest Statement

The authors declare that the research was conducted in the absence of any commercial or financial relationships that could be construed as a potential conflict of interest.
